# Miniature spectrometer based on graded bandgap perovskite filter

**DOI:** 10.1515/nanoph-2024-0112

**Published:** 2024-05-22

**Authors:** Peihan Sun, Xiangmin Hu, Shuhao Yuan, Yanyan Peng, Tingfa Xu, Haizheng Zhong

**Affiliations:** MIIT Key Laboratory for Low-Dimensional Quantum Structure and Devices, School of Materials Science and Engineering, 47833 Beijing Institute of Technology, Beijing, 100081, China; School of Optics and Photonics, 47833 Beijing Institute of Technology, Beijing, 100081, China; CT Corporation Services LLC, Abu Dhabi, 20000, UAE

**Keywords:** ion-exchange, diffusion, graded bandgap perovskite, miniature spectrometer

## Abstract

Miniature spectrometer is powerful tool for scientific research and industrial inspection. Here, we report the fabrication of graded perovskite filters with tunable bandgap and their application in constructing miniature spectrometer. The graded perovskite filters were fabricated using a Finkelstein reaction between *in-situ* formed halogen ion with a preformed MAPbX_3_ film. The graded bandgap of perovskite films can be well tunned from 400 to 750 nm by controlling the volume ratio between 5,5-dimethyl-1-pyrroline *N*-oxide and benzyl chloride(bromide). By combining a deep residual network, graded bandgap perovskite film and commercial CMOS sensor chip, a miniature spectrometer is demonstrated, achieving an accurate spectrum reconstruction (PSNR = 40.749) with monochromatic spectral resolution of 1.31 nm.

## 1 Introduction

Miniature spectrometer is powerful tool for scientific research and industrial inspection [1–6]. Among the reported strategies of miniature spectrometers [1], reconstructive spectrometer, that combine a transmittance spectra tunable filter with a spectrum reconstruction algorithm, is an emerging methodology to construct ultracompact high-performance spectral analysis systems. Without varying its transmissive properties over time like heterojunction filters [7]–[9], or integrating optical microstructure arrays based on Fabry–Pérot etalons [10], [11], photonic crystals [12], metasurface [13], [14], the integrated filter array concept [15] utilizes the tunable but intrinsic optical properties of materials, thus lead to fast response and manufacture simplicity. Bao [16] et al. first has introduced the colloidal quantum dot to construct the filter array. In previous works [17]–[19], the *in-situ* fabricated quantum dot as tunable filters was demonstrated. However, these methodologies usually need to synthesis a series of materials and fabricate each component of the filter array one by one, which are time-consuming and expensive. By contrast, fabricating filter with graded-spectral material can reduce fabrication steps and cost (Table S1). Therefore, there is an intensive need to develop a methodology for fabricating a filter material with graded spectra.

As an ionic semiconductor material with the structure of ABX_3_, perovskite can achieve continuous changes in bandgaps by adjusting the ratio of X-site halogen through ion exchange [20–27]. In recent years, ion exchange of perovskites has been intensively investigated, and several strategies has been proposed to fabricate graded perovskite including solid-gas ion exchange [28], [29], solid-liquid ion exchange [30], [31], self-spreading [32] and composition graded printing [33]. Solid-gas ion exchange method can fabricate composition graded perovskite films by exposing it to some halide gas, and varying the anion exchange level with a temperature gradient. Solid-liquid ion exchange method fabricates graded perovskite film or microwire by dipping pristine perovskite into corresponding halogen solution and then slowly pull it out, while self-spreading method fabricates perovskite graded at the interface by self-spreading and interdiffusion between two perovskite precursor drops. Composition graded printing method uses a binary slot-die coating system, which is suitable for roll-to-roll fabrication but limited to achieve large graded spectral in miniature area due to the slow mixing speed of perovskite precursor. Existing methods for fabricating composition graded perovskite use some electromechanical equipments (for example, a heater, a motor, inkjet printer, etc. as shown in Table S2) thus are energetically unfavorable for scale-up production. Up-to-now, a facile and cost-effective fabrication method to achieve controllable gradient area and wide spectral response for miniature spectrometer is still needed.

In this paper, we developed a facile solid-liquid ion exchange methodology for fabricating graded bandgap MAPbI_3_-MAPbBr_3_–MAPbCl_3_ perovskite film using a Finkelstein reaction between *in-situ* formed halogen ion with a preformed MAPbX_3_ film. The bandgap of perovskite films can be well tunned from 400 to 750 nm by controlling the volume ratio between 5,5-dimethyl-1-pyrroline N-oxide (DMPO) and benzyl chloride(bromide). By combining a deep residual network, graded bandgap perovskite film and commercial CMOS sensor chip, a miniature spectrometer is demonstrated, achieving an accurate spectrum reconstruction (PSNR = 40.749).

## 2 Results and discussion

### 2.1 In-situ study of ion exchange dynamic

In our previous work [23], Finkelstein reaction has been used to successfully conduct Br–Cl ion exchange for perovskite film. However, the ion exchange rate is too low to meet the demand of fast preparation of graded perovskite filters. The Finkelstein reagent was replaced to be benzyl chloride (bromide) which has higher ion exchange reactivity, and free radical trapping agent, 5,5-dimethyl-1-pyrroline *N*-oxide (DMPO) was used as reagent additive to further improve the reactivity. DMPO/benzyl chloride solutions with different volume ratios were prepared, and *in-situ* absorption spectra were recorded since the pristine perovskite thin films immerged into solution.

**Figure 1:**
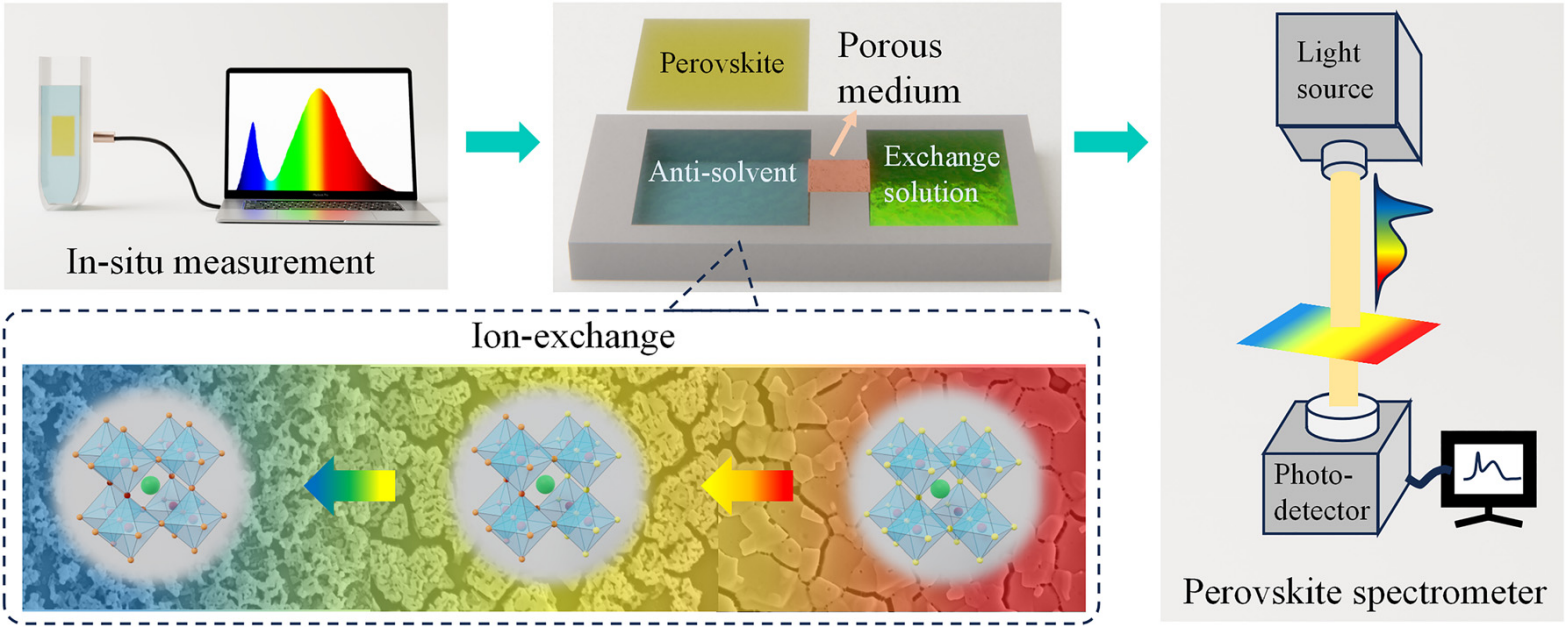
Schematic of graded bandgap perovskite spectrometer research.

As shown in Figure 1, the ion exchange reaction kinetics was studied by *in-situ* monitoring the absorption spectra evolving of the perovskite film in halogen ion source solution. A template was designed to form the anion concentration gradient, which allows ion diffusion from the exchange solution pool to the anti-solvent pools through an ion permeable membrane. Graded bandgap perovskite (GBP) filter can thus be prepared through spatially-graded ion exchange of perovskite film upon the anti-solvent pool. This methodology has great potential for facile and large-scale preparation of graded filters. In addition, a spectrum testing system was integrated using the GBP filter and a CMOS image sensing chip, and a neural network was designed to reconstruct the input spectrum, which demonstrated the novel graded perovskite filter-based spectrometer.

**Figure 2:**
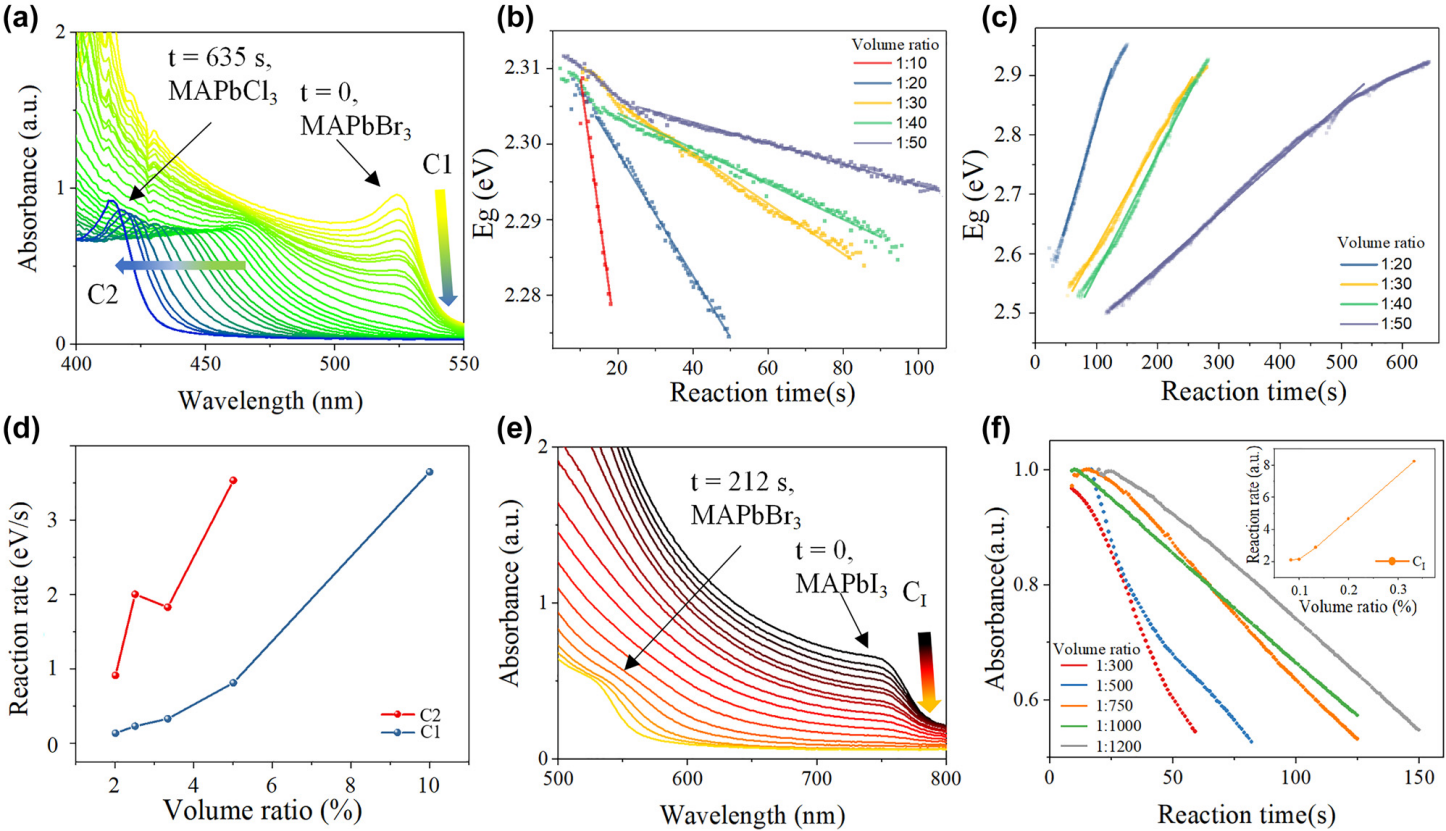
The ion exchange reaction dynamics results. (a) *In-situ* UV–vis spectra evolving with time of MAPbBr_3_–MAPbCl_3_ film. (b) The bandgaps evolving with time of C1 and (c) C2 with different volume ratio. (d) The relation between reaction rate and volume ratio for MAPbBr_3_–MAPbCl_3_ exchange process. (e) *In-situ* UV–vis spectra evolving with time of MAPbI_3_–MAPbBr_3_ film. (f) The absorbance decreasing with time of *C*_I_ and the relation between reaction rate and volume ratio for MAPbI_3_–MAPbBr_3_ exchange process.

Figure 2a shows the absorption spectra evolving with time of MAPbBr_3_ thin films in DMPO/benzyl chloride solution with a volume ratio of 1:50. In Br–Cl exchange process, there are two spectrum evolving characters which can be used to study the exchange rate. The first character is the intensity of the MAPbBr_3_ characteristic absorption onset at ∼525 nm (C1) which gradually decreasing to zero. The second character is the absorption onset at ∼470 nm (C2) which showed up and gradually blue shifting to ∼410 nm. The Tauc plot method [34], [35] (Figure S1) is used to calculate the bandgaps. Figure 2b and c shows the evolving of the bandgap of C1 and C2 with time, respectively. When the volume ratio of DMPO: benzyl chloride is 1:10, C2 is not observed (Figure S2). The phenomenon can be explained to the destruction of perovskite film caused by the ultrahigh Cl^−^ concentration, as demonstrated by the SEM images comparison in Figure S3. The reaction rate of C1 and C2 (Figure 2d) is obtained by fitting the *Eg*–*t* curves in Figure 2b and c, which is approximately linear with the volume ratio of DMPO/benzyl chloride, and the perovskite film will be damaged if the volume ratio is too high.

Figure 2e shows the absorption spectra evolving with time of MAPbI_3_ thin films in DMPO/benzyl bromide solution with a volume ratio of 1:1000. In I–Br exchange process, only the intensity decreasing at the absorption onset of ∼750 nm (*C*_I_) and the appearance of absorption onset at ∼525 nm (*C*_Br_) are observed, while there is no absorption onset among them due to the effect of the solution on the spectral measurement (Figure S4). Thus, the reaction rate cannot be obtained by *Eg*–*t* curve fitting based on Tauc plot method. Instead, the reaction rate can be reflected by the intensity decreasing rate of *C*_I_ (Figure 2f). The reaction rate is also linear with the volume ratio of DMPO/benzyl bromide, and a too high volume-ratio will also destroy the perovskite film (Figure S3). To conclude, there is a compromise between fast exchange rate and film maintenance in both Br–Cl and I–Br ion change processes.

### 2.2 Optimized fabrication of the graded bandgap perovskite filter (GBPF)

**Figure 3:**
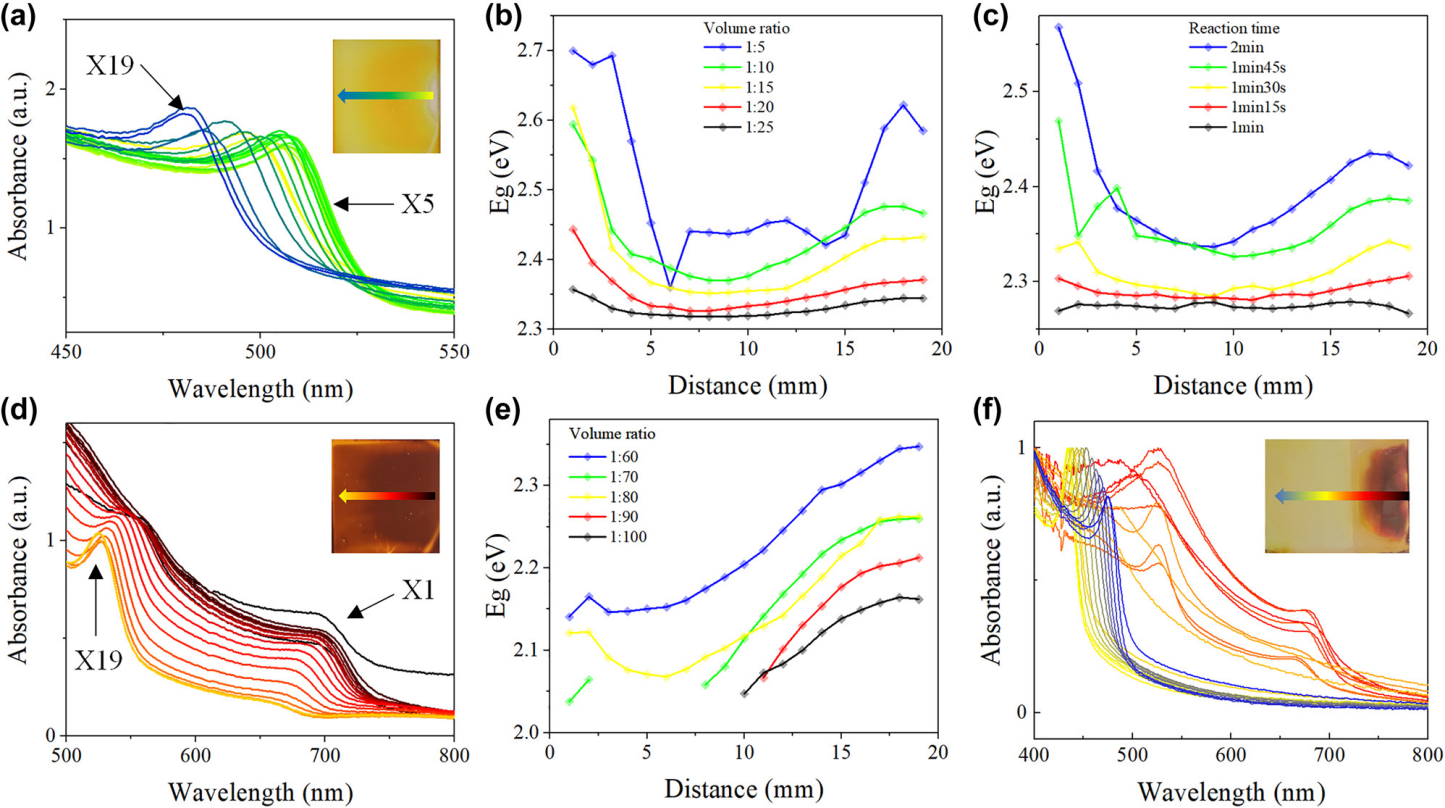
The MAPbI_3_–MAPbBr_3_–MAPbCl_3_ film optimizition. (a) UV–vis spectra measured at different position of MAPbBr_3_–MAPbCl_3_ film. (b) Bandgaps at different position of MAPbBr_3_–MAPbCl_3_ film when exchanged with different volume ratio and (c) different reaction time. (d) UV–vis spectra measured at different position of MAPbI_3_–MAPbBr_3_ film after 2 days. (e) Bandgaps of Br part at different position of MAPbI_3_–MAPbBr_3_ film after 2 days when exchanged with different volume ratio. (f) UV–vis spectra measured at different position of MAPbI_3_–MAPbBr_3_–MAPbCl_3_ film.

By forming diffusion-induced ion concentration gradient, and conducting ion exchange process in the template, well crystallized perovskite film (Figure S5) with spatial bandgap gradient can be fabricated. Due to the requirement of wide spectral response of spectrometer, GBPF fabrication strategy was optimized to achieve wide bandgap distribution, via different exchange solution volume ratio and ion exchange duration experiment. Figure 3a shows the absorption spectra of MAPbBr_*x*_Cl_3−*x*_ thin films measured at different locations after graded ion exchange. The absorption onset of the film changes continuously in the range of 510 nm–460 nm along the ion diffusion direction. The bandgaps at 19 measurement positions (X1–X19) along the diffusion direction when using different volume ratios of DMPO: benzyl chloride solution are shown in Figure 3b. The bandgaps decrease from X1 to X9 and increase from X10 to X19. The range of bandgaps of X1–X9 and X10–X19 broaden from 0.039 eV to 0.340 eV and from 0.028 eV to 0.262 eV, respectively, with the increase of the volume ratio (Table 1). Nevertheless, the bandgaps at different sites show large fluctuation when adopting volume ratio of 1:5, which is due to the perovskite film damage. For compromise between fast exchange rate and film maintenance, the volume ratio of 1:10 was adopted for the follow-up Br–Cl exchange experiments and GBPF fabrication.

**Table 1:** Bandgap range increase with DMPO: benzyl chloride volume ratio.

Volume ratio	Δ*Eg* (X1–X9) [eV]	Δ*Eg* (X10–X19) [eV]
1:5	0.340	0.262
1:10	0.224	0.107
1:15	0.205	0.077
1:20	0.117	0.045
1:25	0.039	0.028

The results showed two directions of ion concentration gradient, because the ion diffusion between two pools is a sophisticated non-linear process ruled by the convection diffusion equation. There are mainly two ion diffusion paths in the 3D-structured template of diffusion experiments. One is high concentration anions diffuse in the solution from ion source pool to anti-solvent pool, which result in the decrease of bandgaps from x1 to x9. The other is that anions move along the bottom surface of the template, which result in the increase of bandgaps from x10 to x19. The counter-intuitive process implies a faster speed for anion to diffuse along the template surface than to diffuse in the solution. Use low wettability template may lead to a linearly varying bandgap which need further hydrodynamics simulations and/or experiments.

Figure 3c shows the bandgaps along the diffusion direction after different reaction time. The bandgap range increase significantly as the reaction time increases from 1 min to 2 min (Table 2). The bandgap range remains if further increasing the exchange time to 2.5 min (Figure S6), accompany with an overall bandgap blueshift. The Cl^−^ concentration gradient faded at ∼2 min, the total reaction time of 2 min was adopted for the follow-up exchange experiments.

**Table 2:** Bandgap range increase with reaction time.

Reaction time [s]	Δ*Eg* (X1–X9) [eV]	Δ*Eg* (X10–X19) [eV]
120	0.231	0.098
105	0.143	0.061
90	0.050	0.058
75	0.023	0.025
60	0.004	0.007

The absorption spectra of the newly exchanged MAPbI_*x*_Br_3−*x*_ film at different positions are shown in Figure S7a, the bandgap (*P*_I_) decreases first and then increases along the diffusion direction, which is similar to Br–Cl exchange process. The bandgap range also broadens with the increase of volume ratio (Figure S7b). However, two absorption onsets (*P*_I_ and *P*_Br_) of MAPbI_*x*_Br_3−*x*_ film were observed after two days storage in ambient condition (Figure 3d), which is due to phase separation. The bandgaps along the diffusion direction when using different volume ratios of DMPO: benzyl bromide solution is shown in Figures 3e (*P*_Br_) and S8 (*P*_I_), in which the missing points are Tauc plot extraction failures due to extremely low *P*_Br_ intensity. The *P*_Br_ related bandgap range increase from 0.163 eV to 0.206 eV with the volume ratio, while the *P*_I_ related bandgap range does not increase significantly. In Figure S8, the bandgaps of *P*_I_ at different sites show large fluctuation when adopting volume ratio of 1:60 due to perovskite film damage. For both fast exchange rate and film maintenance consideration, the volume ratio of 1:70 was adopted for GBPF fabrication.

Based on the bandgap spatial distribution optimization, the MAPbI_3_–MAPbBr_3_–MAPbCl_3_ thin film filter was fabricated through a two-step template ion exchange strategy (detailed in **experimental section:** *Graded perovskite filter fabrication*). The absorbance spectra of the as fabricated GBPF are shown in Figure 3f, which basically covering the whole visible wavelength domain of 400∼750 nm. Further, the absorption spectrum of the prepared perovskite filter hardly changed after stored more than one month in ambient condition (Figure S9), preliminary indicating good stability. However, the perovskite is known to its instability when exposed to humidity, high temperature and light [36]–[38]. The further encapsulation methodology [39], [40] is needed for practical production. The key fabrication parameters in the ion diffusion and exchange process, including ion concentration and reaction time, can be strictly controlled by automation equipments, which lead to the scale-up production of low-cost bandgap graded filter in good reproducibility.

### 2.3 The spectrum reconstruction of graded perovskite filter

**Figure 4:**
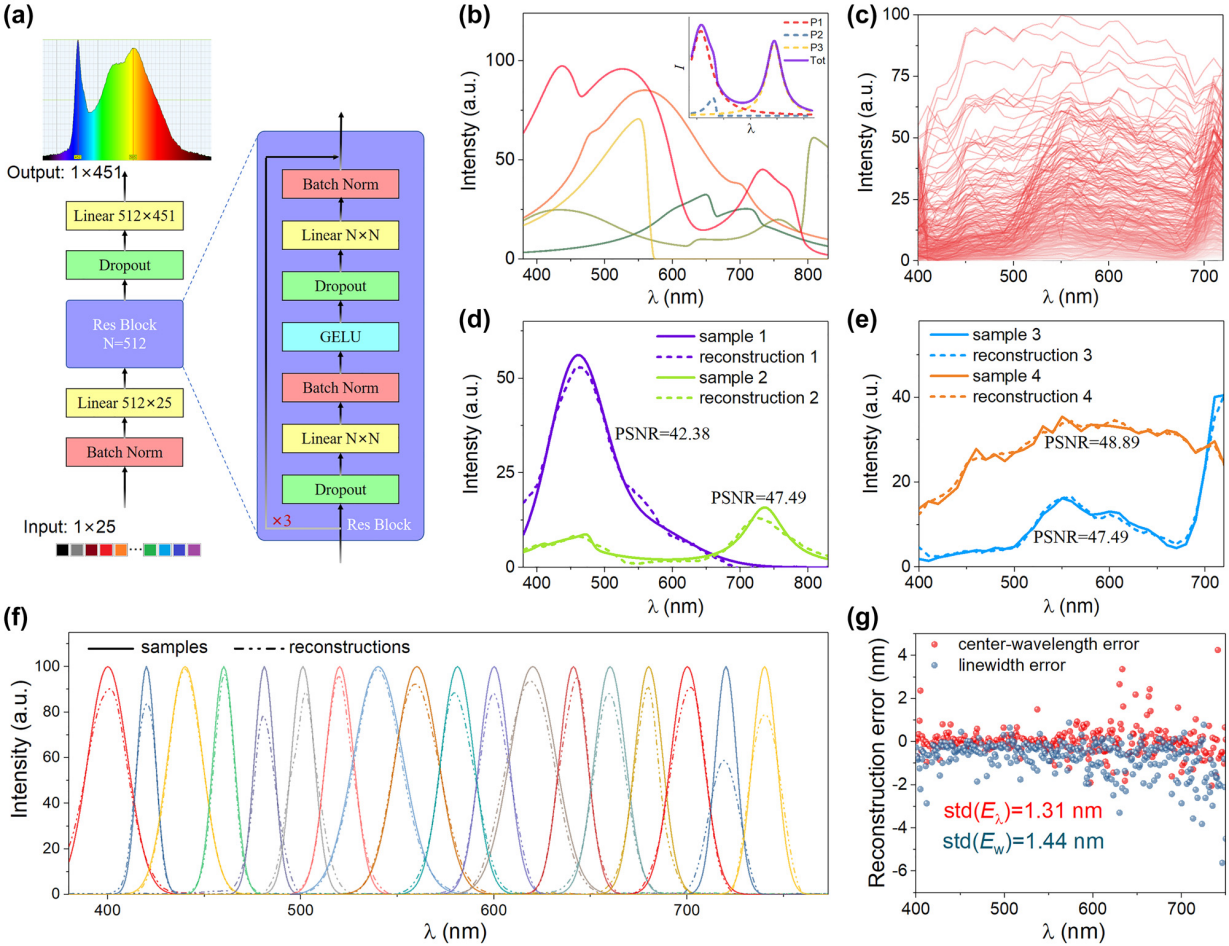
The neural network architecture and the performance of graded perovskite filter. (a) The designed residual network containing three linear layers inserted with two residual blocks. (b) Five synthetic spectra instances. The inset shows the exemplary spectra composed of two Gaussian distribution functions and one asymmetric Lorentzian distribution function. (c) All 322 real spectra selected from a public hyperspectral image (HSI) dataset. The exemplary reconstruction results for (d) synthetic broadband spectra, for (e) HSI spectra and for (f) monochromatic spectra. (g) The reconstruction errors of the center-wavelength and linewidth for the monochromatic spectra.

To evaluate performance in terms of the spectral reconstruction of the as fabricated GBPF, we designed a residual network (Figure 4a), which contains three linear layers inserted with two residual blocks. An input spectral dataset containing 4000 synthetic spectra (3000 broadband and 1000 monochromatic curves) and 322 real spectra were prepared. Five spectra instances are showed in Figure 4b and an exemplary spectra synthesized from Gaussian, Lorentzian curves with simple asymmetric variation [41] is showed as the inset. Figure 4c plots all the 322 real spectra selected from a public hyperspectral image (HSI) dataset [42], [43]. A spectrum testing system was integrated using a parallel light source, the graded perovskite filter, and CMOS image sensing chip, and the graded perovskite filter using transmitted illumination were photographed in darkroom (Figure S10). Twenty-five pixels along the bandgap-graded direction were used to reconstruct the input spectra of the lamp. While it is impossible to collect enough real samples for network training, the pixel signals were simulated by the following formula.

$$P_{i}=\text{CRF}\left(\int_{380}^{830}I\left(\lambda \right)*T_{i}\left(\lambda \right)*\text{QE}\left(\lambda \right)*d\lambda ,E\right)$$

where CRF is the camera response function obtained with Paul’s method [44] (Figure S11), *I*(*λ*) is the input spectrum (the target spectrum to be reconstructed), *T*_*i*_(*λ*) is the transmittance of the graded perovskite filter at position *i* as showed in Figure 3c, QE(*λ*) is the quantum efficiency of the CMOS chip (Figure S12a), and *E* is the time of exposure when photo-taking. The simulated 25 pixel signals match well with the measured pixels (Figure S12b), which implies the effectiveness of using the simulated pixel signals for neural network training.

For each input spectrum, three different exposure times were adopted to obtain the simulated pixel signals. Therefore, ∼13,000 samples with the original spectra curves and the corresponding pixel signals were prepared for spectral reconstruction training. After 20,000 epochs of training, the mean absolute error (MAE) was 1.769 and the average peak signal-to-noise ratio (PSNR) was 40.749. Figure 4d and e show the comparison of input spectra with reconstructed ones for synthetic spectra and HSI spectra, respectively. The constructed spectra well match their input spectra, implying the feasibility of our graded perovskite filter strategy. Figure 4f shows the reconstructed results for a series of monochromatic spectra. The peak positions range from 400 to 750 nm at a step of 20 nm. The standard deviation of the reconstruction errors for the center wavelength is 1.31 nm which outperforms the reported results of 80 nm in ref. [29] and comparable with that of 1 nm in ref. [45], as shown in Figure 4g.

The bandgap graded perovskite filter can be considered as a long wave pass filter array and the high correlation coefficients of the transmittance variance among different site of the filer restricts the spectral resolution. According to our spectral resolution analysis, the theoretical spectral resolution would be as high as 0.05 nm if we take enough transmission signals of different sites of bandgap graded perovskite filter. The key factor that restricts the spectral resolution is the number of different spectral response filter components. The spectral resolution could be estimated as (750–400)/512 = ∼0.68 nm if we take 512 different sites of signal of the graded bandgap perovskite filter. Practically, after optimizing the diffusion template and the fabrication parameters, an appropriately sized bandgap-graded perovskite filter can be directly adhesive onto the CMOS/CCD chip to achieve the highest spectral resolution.

## 3 Conclusions

In conclusion, the feasibility of preparing perovskite spectrometer based on the bandgap-graded perovskite filter has been demonstrated. *In-situ* monitoring the ion exchange process showed that the reaction rate is proportional to the volume ratio of DMPO to benzyl chloride(bromide) solution, and there is an optimal volume ratio to balance fast reaction rate and film maintenance in both Br–Cl and I–Br ion exchange processes. The MAPbI_3_–MAPbBr_3_–MAPbCl_3_ graded bandgap (range from 400 nm to 750 nm) filter with spatial gradient of the transmission spectrum was fabricated by two-step ion exchange strategy. The graded bandgap perovskite spectrometer was demonstrated through spectral sensing test and high precision spectrum reconstruction using neural network. In view of the selection diversity of perovskite, exchange solution material and further optimization of template, as well as the ease to volume production, this work will play a crucial part in application fields like marine scientific research or lab-on-chip, wherever a portable spectrometer is required.

## 4 Experimental section

### 4.1 Materials

All the solvents and chemical reagents used were obtained commercially and used without further purification. Methylamine bromide (MABr, 98 %), methylamine iodide (MAI, 98 %), lead bromide (PbBr_2_, 99.0 %), lead iodide (PbI_2_, 99.99 %), were purchased from Aladdin Biochemical Technology Co., Ltd. *N*, *N*-Dimethylformamide (DMF, Analytical Reagent [AR]), Dimethyl sulfoxide (DMSO, [AR]) and toluene (AR) were purchased from Beijing Tong Guang Fine Chemicals Company. Benzyl chloride (98 %) and benzyl bromide (96 %) were purchased from Meryer (Shanghai) Chemical Technology Co., Ltd. 5,5-dimethyl-1-pyrroline *N*-oxide (DMPO, 98 %) was purchased from Energy-Chemical Co., Ltd.

### 4.2 Preparation of perovskite precursor

MAPbBr_3_ and MAPbI_3_ solutions were prepared by dissolving PbBr_2_ and MABr, PbI_2_ and MAI in DMF: DMSO mixed solvent, respectively. MAPbBr_3_ solution was prepared by dissolving 1.2 mmol PbBr_2_ and 1.2 mmol MABr in 1 mL DMF/DMSO (v/v, 7:3) mixed solvent. MAPbI_3_ solution was prepared by dissolving 0.8 mmol PbI_2_ and 0.8 mmol MAI in 1 mL DMF/DMSO (v/v, 7:3) mixed solvent. The solutions were stirred at room temperature for 6 h before using.

### 4.3 Preparation of perovskite thin film

Glass substrates were cleaned by sequential sonication in water, ethanol, acetone, and isopropyl alcohol for 15 min each and then dried with a nitrogen gas gun. The cleaned glass substrates were further treated with an oxygen plasma treatment (PCA06-2, MTI Corporation) for 3 min. MAPbBr_3_ film was fabricated via a “one-step” process on the glass substrates. 100 μL of the prepared MAPbBr_3_ solution was dripped onto the cleaned glass substrate, then spin-coating at 5000 r.p.m. for 30 s, dripped 300 μL of toluene as anti-solvent at the 15th second, and then annealed for 10 min at 100 °C. The whole process was operated in glove box. MAPbI_3_ film was fabricated as follows: 100 μL of prepared MAPbI_3_ solution was dripped onto the cleaned glass substrate, then spin-coating at 500 r.p.m. for 5 s and 3000 r.p.m. for 40 s, dripped 500 μL of Chlorobenzene as anti-solvent at the 20th second and then annealed for 10 min at 100 °C. The whole process was operated in glove box.

### 4.4 In-situ measurement of ion exchange reaction rate

Place the cuvette with 2 mL of ion exchange solution between the in the detector and light source, then record base line. The prepared perovskite film was cut into size of 0.8 mm × 25 mm, place the film vertically in the solution, after reaction begin, record the spectra data every 0.5 s.

### 4.5 Graded perovskite filter fabrication and characterization

The ion exchange solution was prepared by mix benzyl chloride(iodide) and DMPO with different volume ratio. The solution was placed in atmosphere for 5 h before using. The template was cleaned in water and ethanol and then dried with a nitrogen gas gun. Four layers of thin makeup cotton were placed in the middle groove to separate solution. Then 800 μL of toluene was added to the left pool, and the fabricated perovskite thin film was placed upside down to guarantee the surface of the film to fully contact the toluene. After adding 700 μL of ion exchange solution in the right pool, the solution diffusion process and ion exchange process began in the meantime. To terminate the ion exchange process, the perovskite film was moved from the upward side of left pool to a beaker with 20 mL of pure toluene. The perovskite film was immersed in toluene for 15–20 s to terminate the reaction and wash out the remaining ion exchange solution. Two step template ion exchange strategy: when fabricating the MAPbI_3_–MAPbBr_3_–MAPbCl_3_ film, the operation above was performed twice. At the first time, ion exchange MAPbI_3_ film by Benzyl Iodide, then whole exchange the 1.5 mm region in the other side into MAPbBr_3_ by immerging the film into DMPO: Benzyl Iodide: with a v/v ratio of 1:250 for 6 min. The second operation performed Br–Cl exchange in the 1.5 mm region to obtain I–Br–Cl film. The UV–vis measurement was used an AvaSpec-HSC1024*58TEC-EVO. The exchanged film was fixed on the translation stage along the test direction, and the absorption spectra was recorded every 1 mm. The SEM measurement was used an S8230 (Hitachi, Ltd.). The XRD measurement was used a Smartlab SE (Rigaku Corporation).

## Supplementary Material

Supplementary Material Details
