# Supplementary material for: Miniature spectrometer based on graded bandgap perovskite filter
Source: Nanophotonics. 2024 May 22;13(18):3599–607. doi: 10.1515/nanoph-2024-0112 (PMC11501710; doi:10.1515/nanoph-2024-0112)
Supplement: Supplementary file 1 — Supplementary Material Details [file j_nanoph-2024-0112_suppl_001.docx]

Supporting Information

**Miniature Spectrometer Based on Graded Bandgap Perovskite Filter**

*Peihan Sun, Xiangmin Hu^*^, Shuhao Yuan, Yanyan Peng, Tingfa Xu, Haizheng Zhong*

Table S1. Comparison of filter methodology for reconstructive spectrometer.

| Work | Principle of filter transmittance modulation | | Feature | |  |
| --- | --- | --- | --- | --- | --- |
| *Advanced Materials*, Vol. 34, pp. 2200221, 2022 | 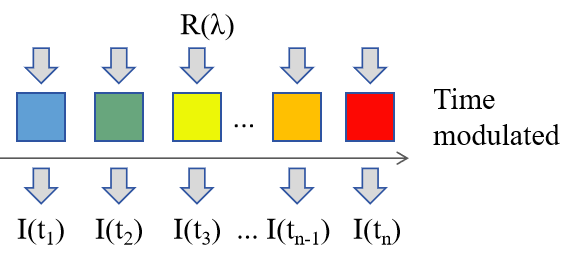 | | - Use only one photodetector - Time modulated spectral response | |  |
| *Nature*, vol. 523, pp. 67-70, 2015 (First work) | | 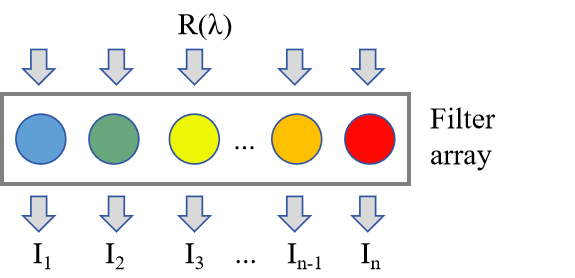 | | - Synthesis materials in multistep - Fabricate filter array in multistep - Limited amount of filter components by multistep fabrication procedure | |
| *Light: Science & Applications*, vol. 9, pp. 73, 2020 (Our previous work) | |  |  |  |  |
| *Adv. Opt. Mater.*, vol. 9, pp. 2100376, 2021 (Our previous work) | |  |  |  |  |
| *ACS Applied Materials & Interfaces*, vol. 15, pp. 7129-7136, 2023 | |  |  |  |  |
| *Adv. Mater.*, vol. 32, pp. 1908108, 2020  *Nano Research*, vol. 16, pp. 10256-10262, 2023  *Adv. Mater.*, vol. 34, pp. 2108408, 2022 | 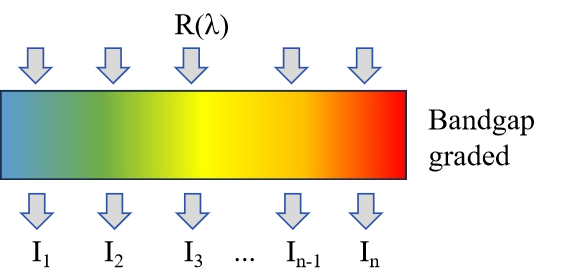 | | - Fabricate spatial varying filter components in one step - Facile and fast fabrication - Large amount of filters with different spectral responses | |  |

**Table S2**. Comparison of fabrication methodology, spectrum reconstruction algorithm, and spectrometer performance for bandgap graded perovskite.

| Work | Bandgap graded perovskite fabrication | Fabrication speed | Spectral range | Spectrometer resolution | Reconstruction algorithm |
| --- | --- | --- | --- | --- | --- |
| *ACS Applied Materials & Interfaces*, vol. 15, pp. 7129-7136, 2023 | 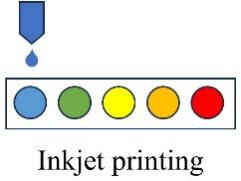 | multistep | 450-700 nm | 10 nm | Gaussian basis function fitting optimized by regularized non-negative  least squares |
| *Adv. Mater.*, vol. 32, pp. 1908108, 2020 | 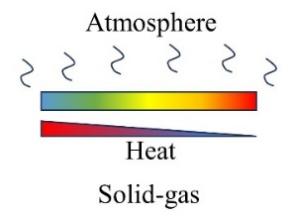 | ~ 20 min | 410-750 nm | 80 nm | Without reconstruction |
| *Adv. Mater.*, vol. 34, pp. 2108408, 2022 | 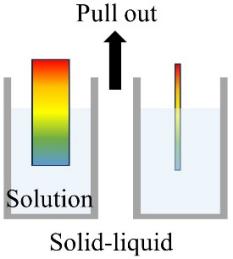 | < 10 s | 450-790 nm | 25 nm | Gaussian basis function fitting optimized by Tikhonov regularization |
| *Light Sci Appl*, vol. 9, pp. 162, 2020 |  | < 10 s | Without spectrometer demonstration | | |
| *Nano Research*, vol. 16, pp. 10256-10262, 2023 | 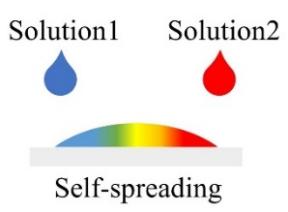 | ~ 100 s | 455-760 nm | 19.8 nm | Gaussian basis function fitting optimized by Tikhonov regularization |
| *Communications Materials*, vol. 3, pp. 13, 2022 | 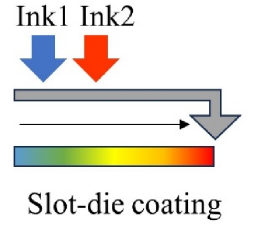 | < 60 s | Without spectrometer demonstration | | |
| This work | 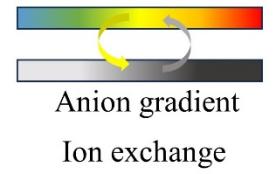 | ~ 120 s | 450-750 nm | ~1 nm | Residual neural network |


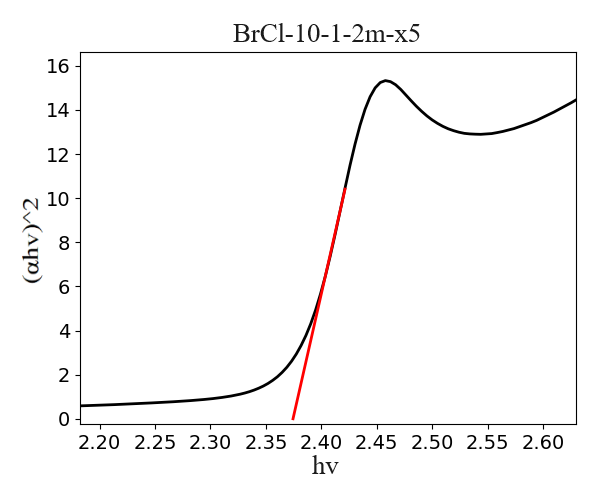


**Figure S1.** Calculation of bandgap for position X5 at MAPbBr_3_- MAPbCl_3_.

The Tauc plot method is a method for calculating the band gap width of semiconductors, which is based on the formula proposed by Tauc Davis and Mott et al.

$${(\alpha h\nu)}^{1/n}=A(h\nu-Eg)$$

Where α is the light absorption index, *h* is Planck's constant, $\nu$ is the frequency, and *Eg* is the semiconductor bandgap. The exponential *n* is dependent on the type of semiconductor, with *n*=1/2 for direct bandgap semiconductors and *n*=2 for indirect bandgap semiconductors. Perovskites are direct bandgap semiconductors, so take *n*=1/2. As shown in Fig. S1, ${(\alpha h\nu)}^{2}$ is taken as the vertical coordinate and $h\nu$ as the horizontal coordinate. The intercept between the line segment and the x-axis is taken as the bandgap width.


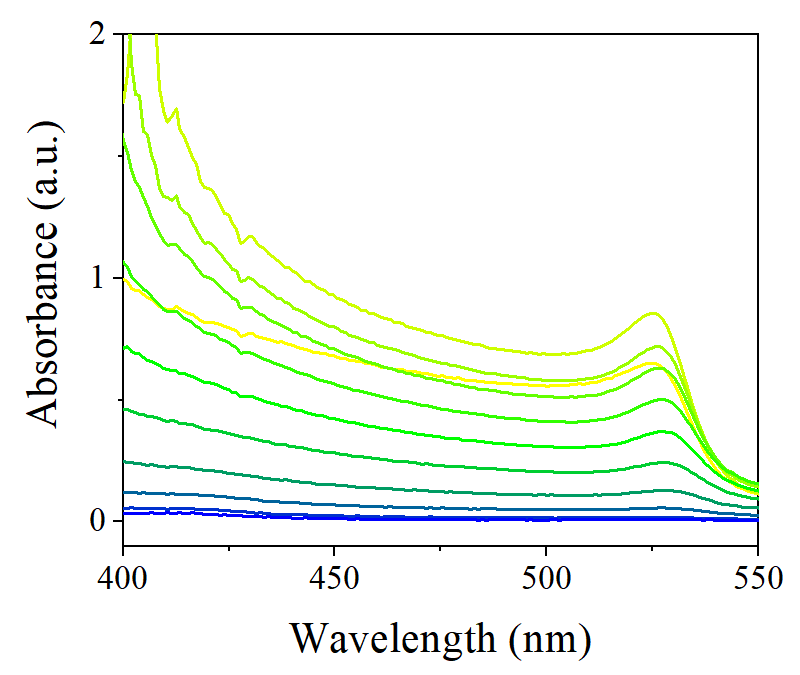


**Figure S2.** In-situ measured UV-vis spectra of Br-Cl exchange process with a DMPO: benzyl chloride volume ratio of 1:10.


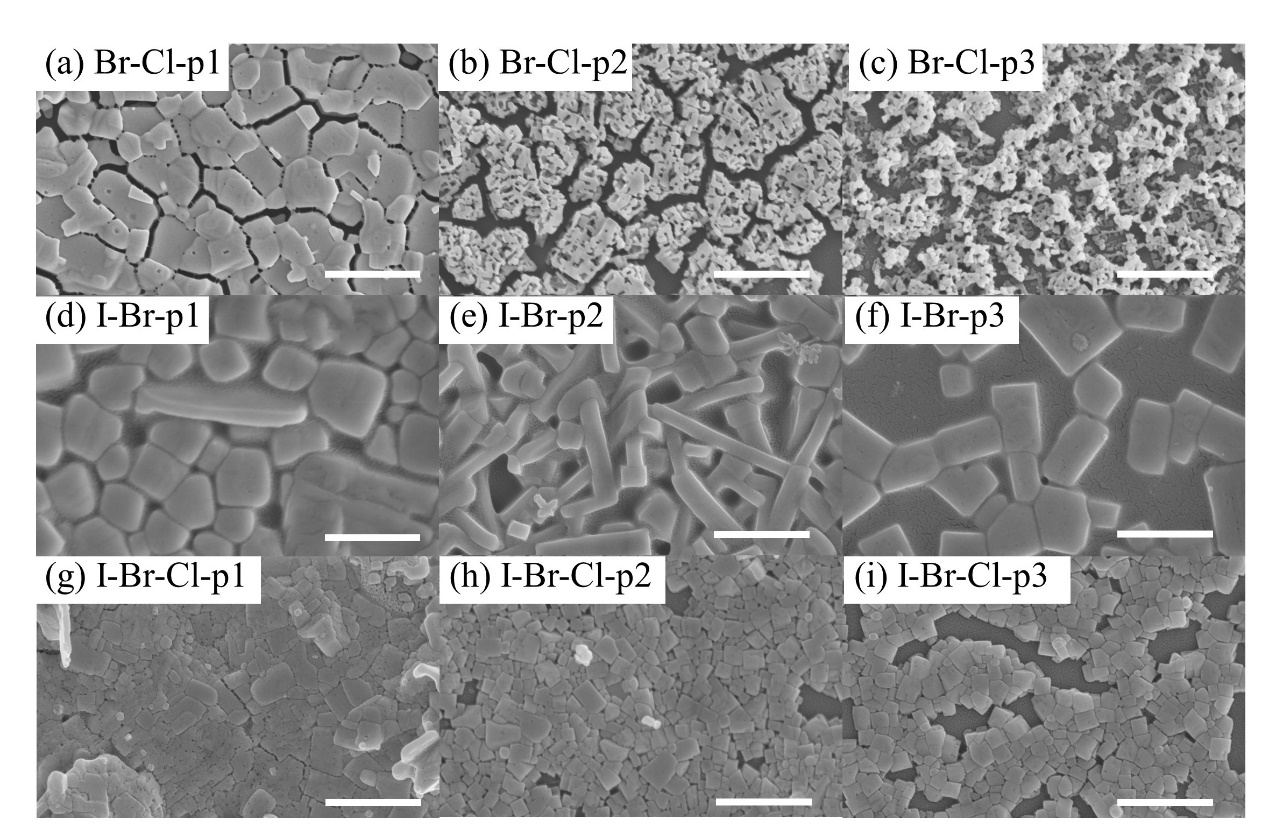


**Figure S3**. SEM image of different position of a-c) MAPbBr_3_- MAPbCl_3_ film, d-f) MAPbI_3_- MAPbBr_3_ film and g-h) MAPbI_3_- MAPbBr_3_- MAPbCl_3_ film, the scale bar equals to 1 um.


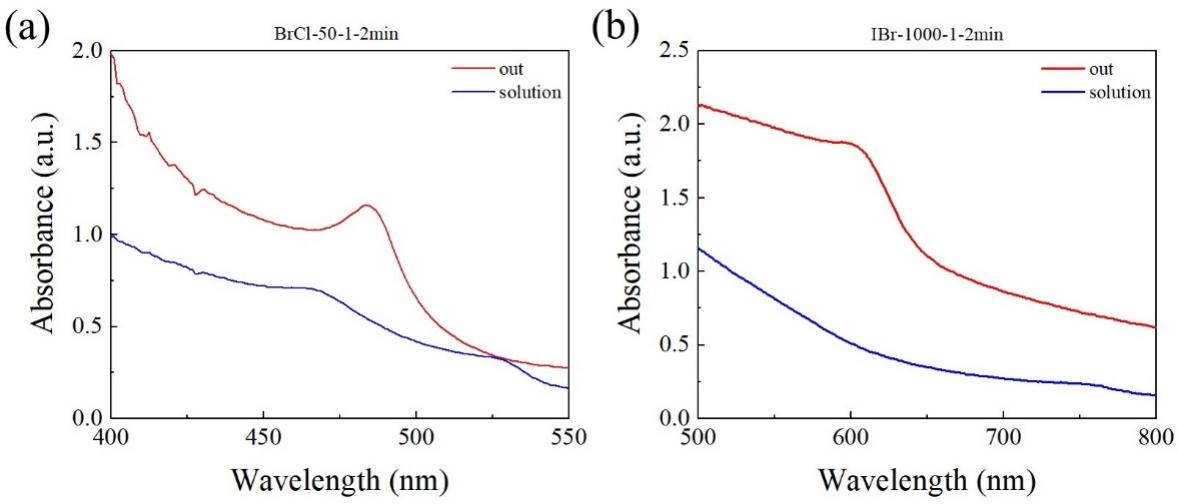


**Figure S4.** Comparation of UV-vis spectra in and out of solution after 2min of reaction during a) MAPbBr_3_- MAPbCl_3_ and b) MAPbI_3_- MAPbBr_3_ exchange process).

The MAPbI_3_ film was removed from the solution after 2min and then immersed in toluene anti-solvent to terminate the exchange. The absorption spectrum obtained was shown in Figure S4b. The results measured outside the solution have a clear absorption onset at ~625 nm, indicating the exchange reaction was processing but cannot be measured in the solution because of the effect of the exchange solution. Similarly, the he absorption spectrum of MAPbBr_3_ film (Figure S4a) under same condition also showed an absorption onset at ~485 nm, which could not be measured in solution. Therefore, the evolving of absorption onset persisted during ion exchange reaction, the observed spectrum can be used to study the reaction kinetic.


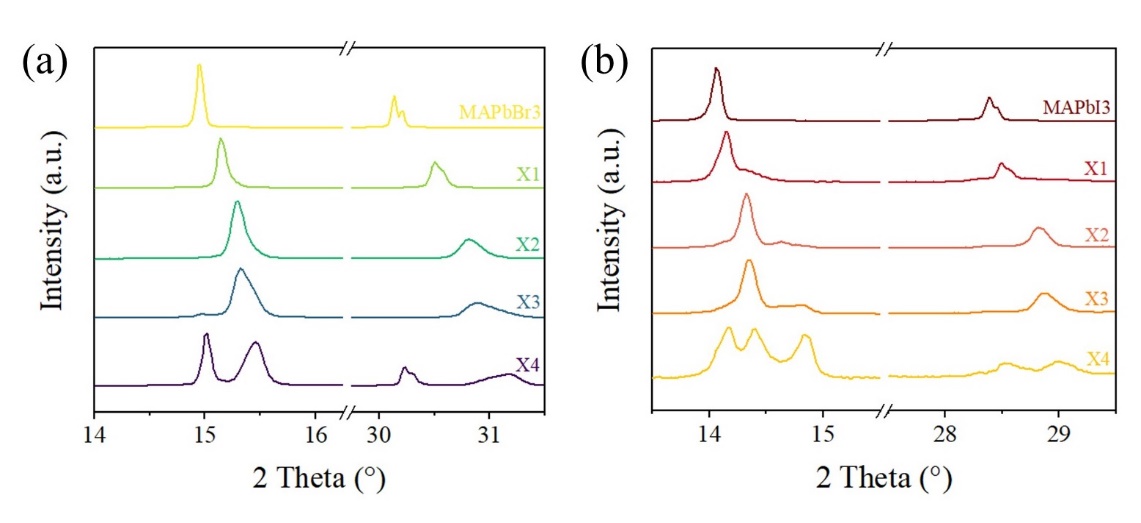


**Figure S5.** XRD spectrum at different position of a) MAPbBr_3_- MAPbCl_3_ film and b) MAPbI_3_- MAPbBr_3_ GBPF.


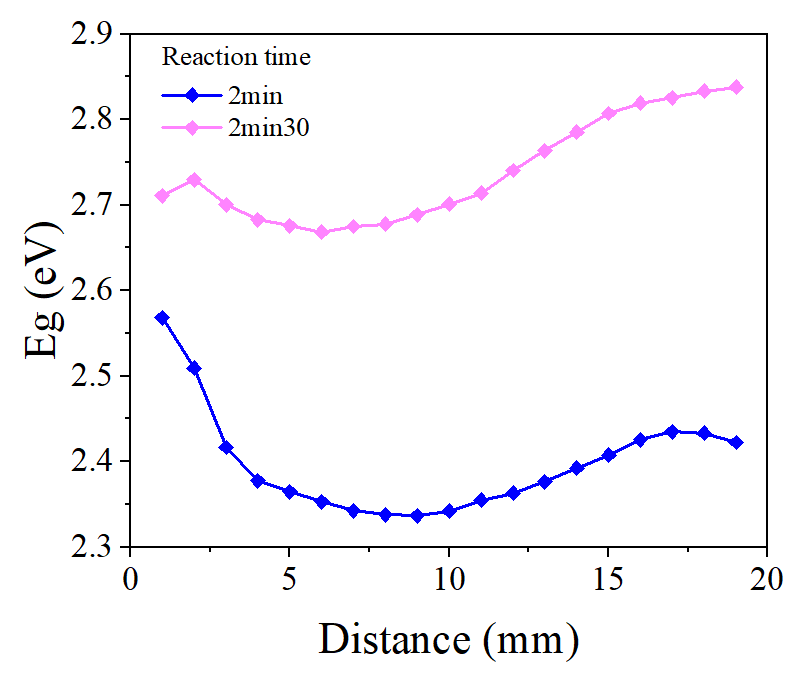


**Figure S6.** Comparation of bandgaps at different position when exchanged with volume ratio of 10:1, reaction time of 2 min and 2 min 30 s of MAPbBr_3_- MAPbCl_3_ film.


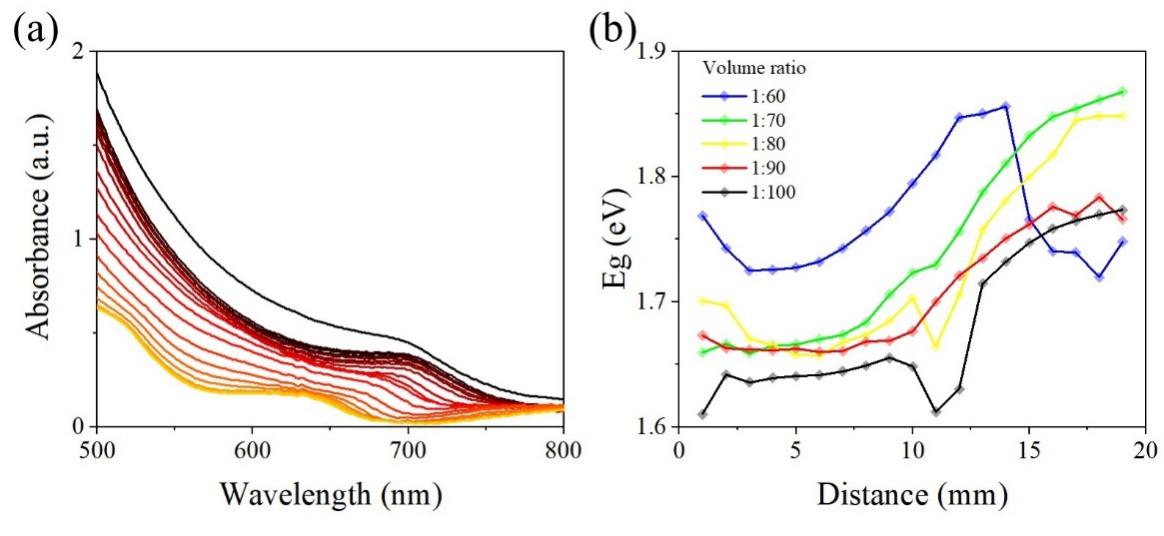


**Figure S7.** a) UV-vis spectra measured at different position in the graded region of MAPbI_3_- MAPbBr_3_ with volume ratio of 70:1, reaction time of 2min. b) Bandgaps at different position when exchanged with different volume ratio of MAPbI_3_- MAPbBr_3_ film).


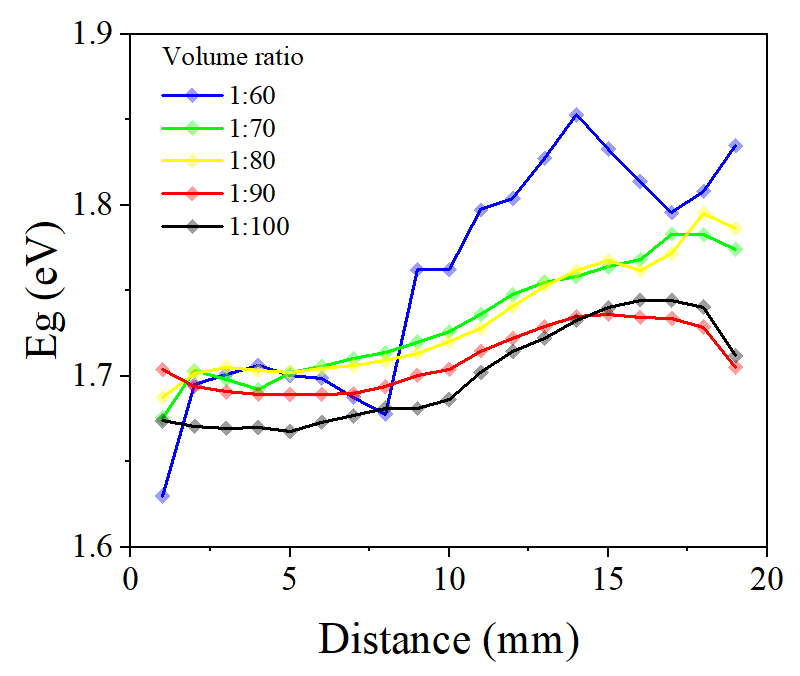


**Figure S8.** Bandgaps of I part at different position when exchanged with different volume ratio of MAPbI_3_- MAPbBr_3_ film after 2 day.


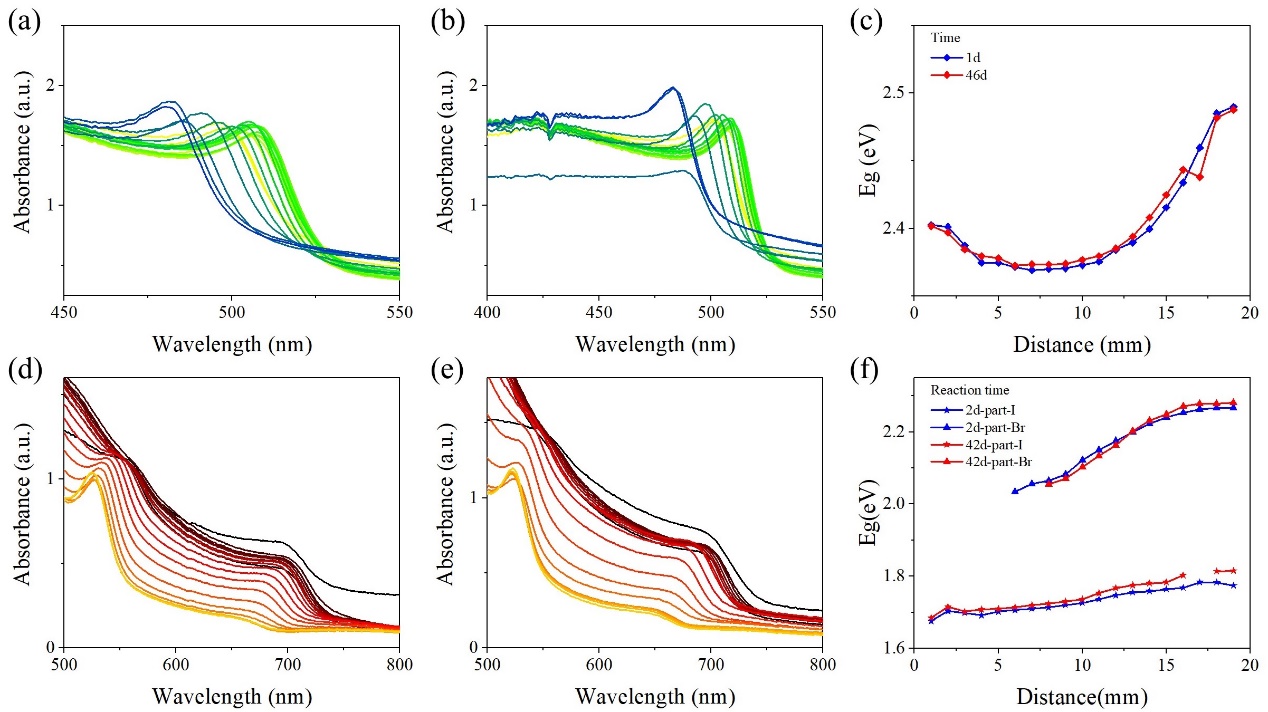


**Figure S9.** a) UV-vis spectra measured at different position of MAPbBr_3_- MAPbCl_3_ GBPF after 1 day and b) 46 days with volume ratio of 10:1, reaction time of 2min. c) Comparation of bandgaps at different position of MAPbBr_3_- MAPbCl_3_ GBPF after 1 day and 46 days. d) UV-vis spectra measured at different position of MAPbI_3_- MAPbBr_3_ GBPF after 2 days and e) 42 days with volume ratio of 70:1, reaction time of 2min. f) Comparation of bandgaps at different position of MAPbI_3_- MAPbBr_3_ after 2 days and 42 days.


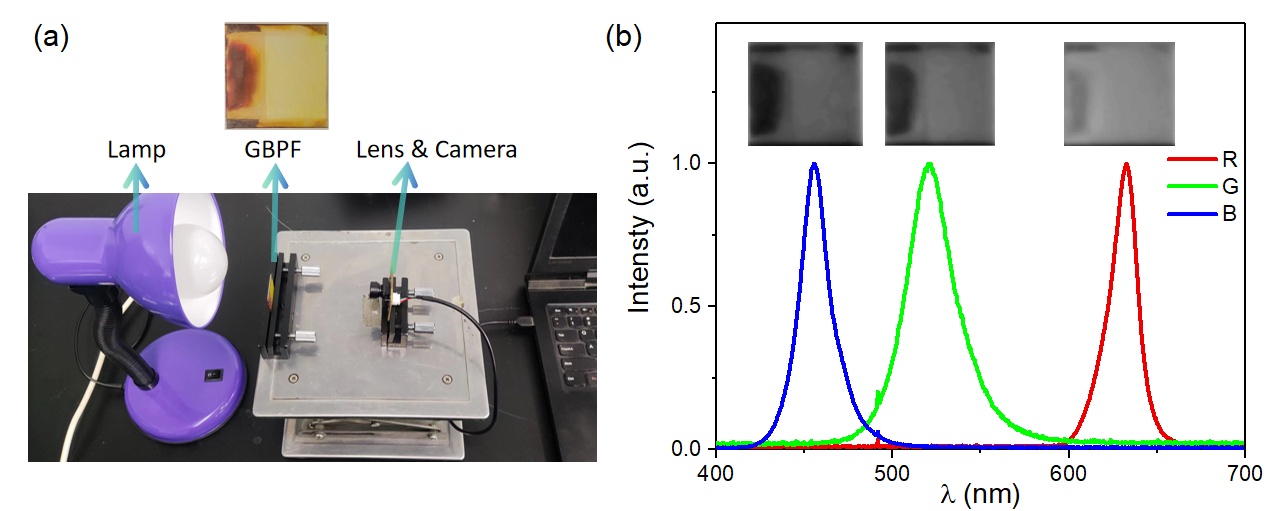


**Figure S10.** The spectrum testing system. a) Spectrum testing setup integrating a lamp, the graded bandgap perovskite filter (GBPF), and a CMOS image sensing chip. b) The photographs of graded perovskite filter in darkroom using transmitted illumination and the corresponding light source spectra.


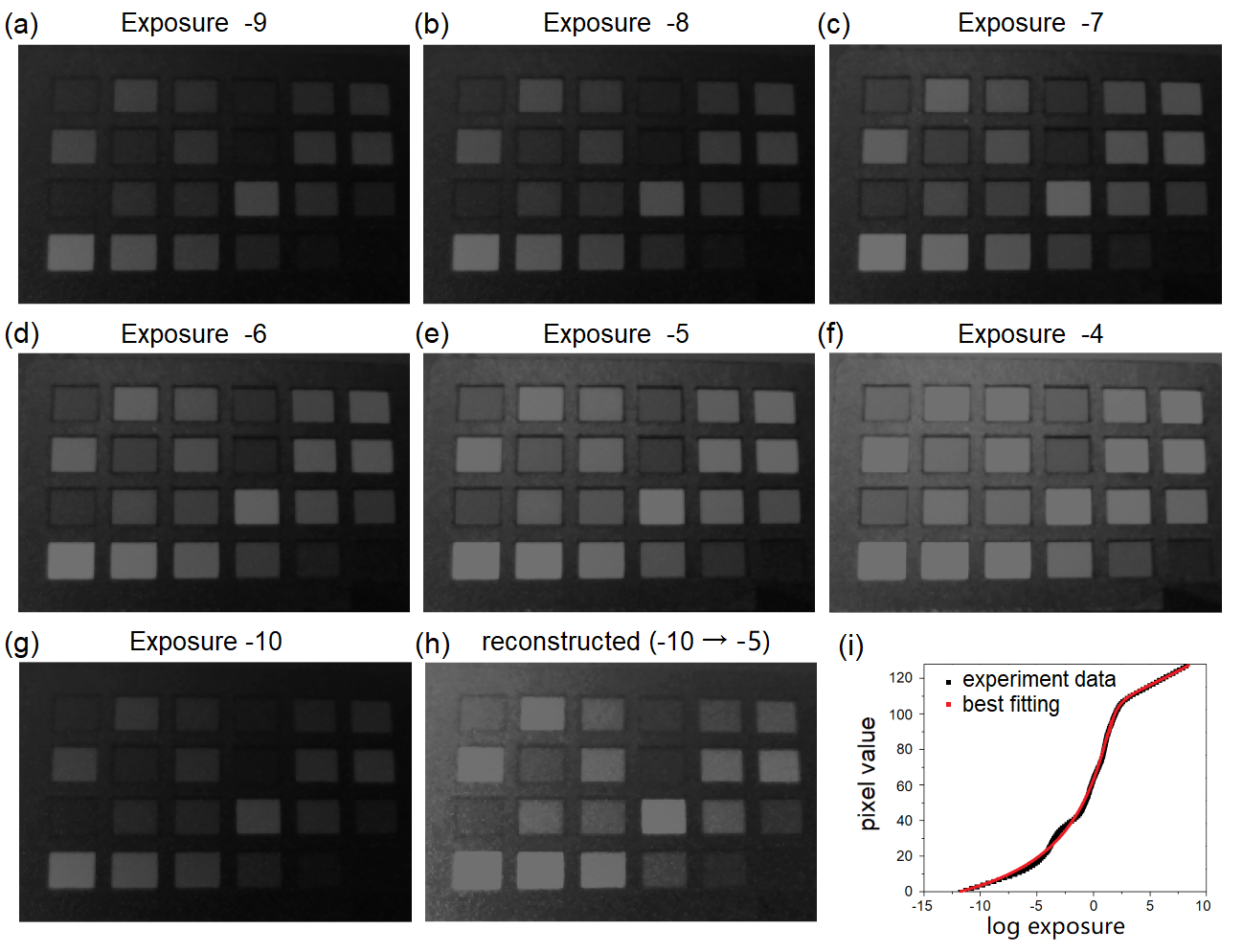


**Figure S11.** The reconstruction of the camera response function uses differently exposed photographs (from -10 to -4). a-g) The photographs of an X-Rite ColorChecker uses differently exposure mode from -10 to -4. h) The reconstructed photograph from using exposure mode -10 to using that of -5, which matches the photo e) well. i) The reconstructed camera response function curve.


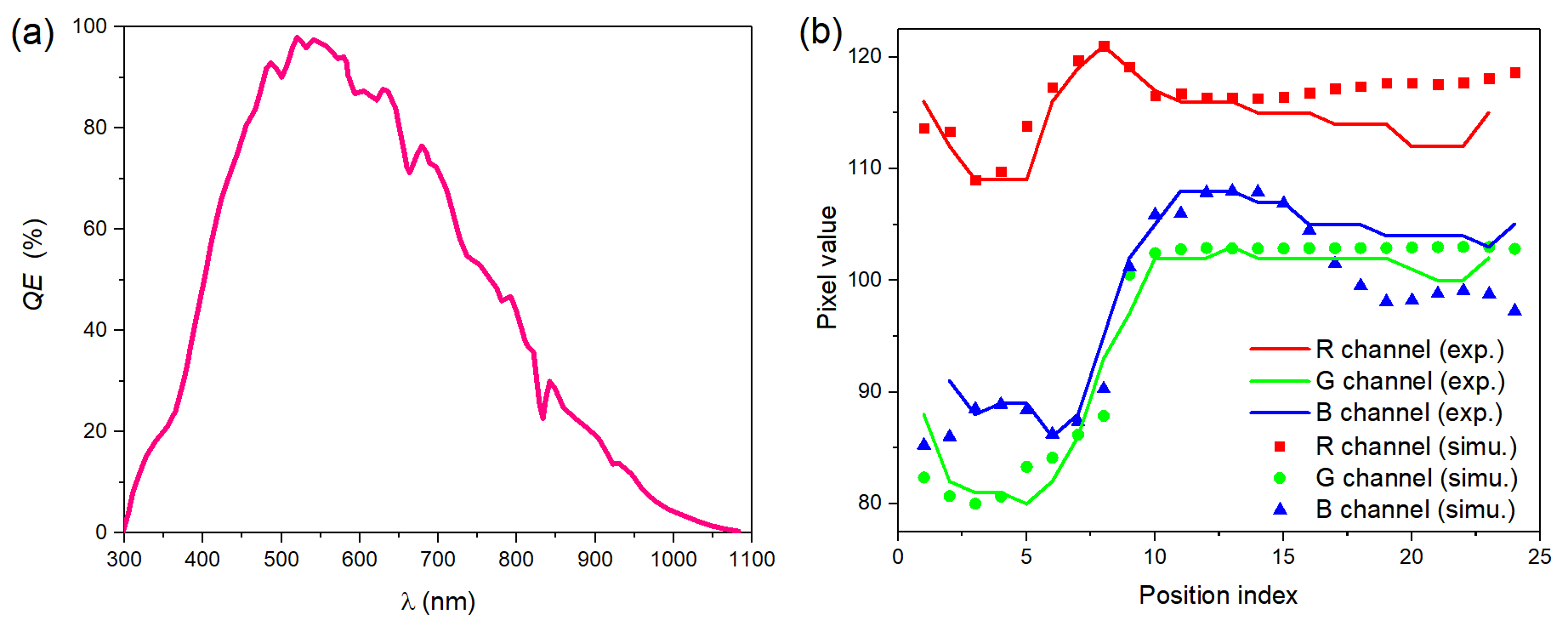


**Figure S12.** The spectrum response curve and pixel simulation result. a) The OV7251 spectrum response curve from the camera datasheet provided by OmniVision company. b) The comparison of experimental (photographs from Fig. S10b) and simulated pixel vales.


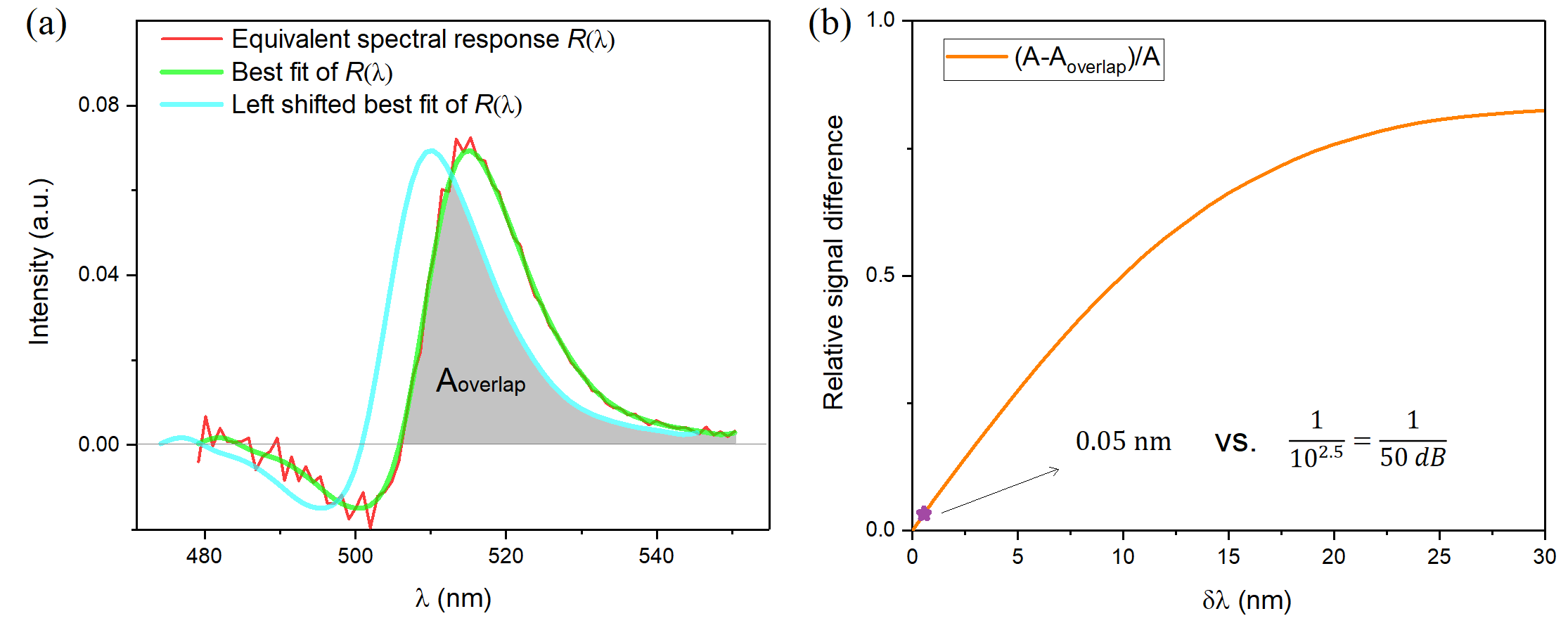


**Figure S13.** The spectral resolution analysis of the spectrometer based on graded bandgap per-ovskite filter. a) The equivalent spectral response function plot with the response over-lap diagram. b) The relative signal difference curve to the center wavelength difference of two adjacent sites on the bandgap-graded perovskite filter.

Considering the transmittance spectra of two sites of the bandgap graded perovskite filter, it is equivalent to express the spectral response function as the first-order derivative of the transmittance spectra. The transmittance signals would be distinguishable if the spectral response function overlaps too much. As the center wavelength of two spectral response function become closer, the corresponding two signal value difference, defined as (A-A_overlap_)/A, where A is the integral of spectral response functions, becomes smaller. The signal will be submerged in noise if the relative signal difference smaller than noise/signal (i.e. 1/SNR). The typical SNR of 50 dB for industrial camera, will allow a 0.05 nm spectral resolution as marked star in Figure S13.
